# Triploid Production from Interspecific Crosses of Two Diploid Perennial *Helianthus* with Diploid Cultivated Sunflower (*Helianthus annuus* L.)

**DOI:** 10.1534/g3.116.036327

**Published:** 2017-02-07

**Authors:** Zhao Liu, Gerald J. Seiler, Thomas J. Gulya, Jiuhuan Feng, Khalid Y. Rashid, Xiwen Cai, Chao-Chien Jan

**Affiliations:** * Department of Plant Sciences, North Dakota State University, Fargo, North Dakota 58108; † United States Department of Agriculture-Agricultural Research Service, Northern Crop Science Laboratory, Fargo, North Dakota 58102; ‡ Plant Science Department, South Dakota State University, Brookings, South Dakota 57007; § Agriculture and Agri-Food Canada, Morden, Manitoba R6M 1Y5, Canada

**Keywords:** *Helianthus*, triploids, genomic *in situ* hybridization (GISH), mitotic analysis, meiotic analysis

## Abstract

Wild *Helianthus* species are a valuable genetic resource for the improvement of cultivated sunflower. We report the discovery and characterization of a unique high frequency production of triploids when cultivated sunflower was pollinated by specific accessions of diploid *Helianthus nuttallii* T. & G. and *H. maximiliani* Schr. Genomic *in situ* hybridization (GISH) analyses indicated that the triploid F_1_s had two genomes from the wild pollen sources and one from the cultivated line. Mitotic chromosome analyses indicated that the frequency of triploid progenies from the crosses of cultivated lines × *H. nuttallii* accession 102 (N102) was significantly higher than those of unexpected polyploid progenies from the crosses of wild perennial species × N102, and no unexpected polyploids were obtained from the reverse crosses. Pollen stainability analysis suggested the existence of a low percentage of unreduced (2*n*) male gametes in some accessions, especially N102 and *H. maximiliani* accession 1113 (M1113), which were generated at the telophase II and tetrad stages of meiosis. The triploid F_1_s could be the results of preferred fertilization of the low frequency of 2*n* male gametes with the female gametes of the cultivated sunflower, due to the dosage factors related to recognition and rejection of foreign pollen during fertilization. The triploids have been used to produce amphiploids and aneuploids. Future studies of the male gametes’ fate from pollination through fertilization will further uncover the mechanism of this whole genome transmission. Studies of the genetic control of this trait will facilitate research on sunflower polyploidy speciation and evolution, and the utilization of this trait in sunflower breeding.

Polyploidy is the presence of two or more complete sets of chromosomes from a single species (autopolyploidy) or two or more species (alloployploidy) in a single organism (Matsushita *et al.* 2012; Mason and Pires 2015). Hybridization and polyploidization are important mechanisms for diversification and speciation during evolution (Storme and Mason 2014). Phylogenetic studies and comparative genome analyses confirmed that most flowering plants have undergone one or more rounds of ancient polyploidy early in their evolutionary history, such as two recent whole genome duplications (named α and β) within the crucifer (Brassicaceae) lineage and one common genome triplication event (γ) within all core eudicots (Jaillon *et al.* 2007; Jiao *et al.* 2011). Up to 70% of angiosperm species are secondary polyploids, which are believed to arise commonly through the production of unreduced gametes (2*n* gametes) resulting from meiotic and premeiotic defects (Brownfield and Köhler 2011; Storme and Mason 2014).

Unreduced gametes are commonly produced by interspecific hybrids, and can also be induced by abiotic and biotic stresses (Mason and Pires 2015). In a comparison of 11 hybrid to 11 nonhybrid angiosperm species, diploid F_1_ hybrids produced 27.52% 2*n* gametes, and the nonhybrids only 0.56%. It was also noted that higher frequencies of polyploidy occurred in perennial taxa capable of vegetative reproduction, and plant families lacking endosperm in mature seeds, such as the Asteraceae, Crassulaceae, Onagraceae, Rosaceae, and Salicaceae (Ramsey and Schemske 1998). Triploidy can result either from the fusion of a 2*n* gamete to a regular reduced gamete (n), with both produced by diploid individuals, or from crosses between diploid and tetraploid individuals. The evolution of polyploids was more likely through a triploid bridge than through other mechanisms (Mason *et al.* 2011). Triploids also could act as vectors for gene flow between diploid and tetraploid populations (Henry *et al.* 2005).

The genus *Helianthus* includes 53 species, *i.e.*, 39 perennials and 14 annuals maintained at the USDA-ARS, North Central Regional Plant Introduction Station (NCRPIS) in Ames, IA (Seiler and Jan 2014). The 14 annual species are diploid (2*n* = 2*x* = 34), and the 39 perennial species include 26 diploid, three tetraploid (2*n* = 4*x* = 68), seven hexaploid (2*n* = 6*x* = 102), and one mix-ploid of either diploid or tetraploid, and two mix-ploids of tetraploid or hexaploid. A large number of sunflower interspecific hybrids have been evaluated since the pioneering work of Heiser and his students in the1940s (Jan 1997). Initial interspecific hybridizations were among wild annual *Helianthus* species and their crosses with cultivated sunflower. Interspecific hybrids involving perennial species followed the establishment of embryo rescue techniques (Chandler and Beard 1983). No unusually abnormal cytological observations have been reported in wild *Helianthus* species, but abnormal meiosis is the norm in almost all the interspecific F_1_ progenies, especially between wild species and the cultivated sunflower. However, a limited number of unreduced gametes can frequently be observed, which could be the driving force of sunflower polyploidization, speciation, and evolution.

Several tetraploid interspecific amphiploids were produced by the authors via colchicine treatment of the F_1_ hybrids followed by intercrossing of heads containing a high frequency of larger pollen grains, assumed to be chromosomally doubled, compared to the smaller pollen grains associated with a haploid set of chromosomes. As a general rule, large pollen grains of interspecific hybrids indicated unreduced gametes which have the potential of producing a low frequency of interspecific amphiploids via sib-pollination without colchicine treatment. This has been confirmed in our lab by backcrossing several interspecific hybrids with cultivated sunflower pollen without emasculation, resulting in progenies having chromosome numbers of 2*n* = 4*x* = 68 or 2*n* = 2*x* + *x* = 51 for diploid hybrids, 2*n* = 6*x* = 102 or 2*n* = 4*x* = 68 for triploid hybrids, or 2*n* = 8*x* = 136 or 2*n* = 5*x* = 85 for hexaploid hybrids, suggesting self-pollination of the unreduced male to female gametes or unreduced female gametes pollinated by the haploid cultivated pollen.

A group of accessions of diploid perennial *Helianthus nuttallii* and *H. maximiliani* collected in the vicinity of Morden, Manitoba, Canada with resistance to *Sclerotinia sclerotiorum* (Lib.) de Bary head rot were pollinated to a cultivated sunflower line to transfer the resistance genes (Jan *et al.* 2007). An unusually high frequency of triploids in the F_1_ progeny was observed. This phenomenon had never been observed in diploid × diploid crosses involving wild perennial *Helianthus* and cultivated lines. These wild accessions were further confirmed to be diploid and had 2*n* = 2*x* = 34 chromosomes. However, as expected for diploid × diploid crosses, all F_1_ progeny were diploid when the wild accessions were used as the female parents. The clear-cut reciprocal differences of producing F_1_ progeny at 2*n* = 3*x* and 2*n* = 2*x* pointed to the normal megasporogenesis of the wild accessions and the likely abnormal microsporogenesis of the male gametes and/or abnormal fertilization process. This study focuses on the confirmation of this novel discovery with additional interspecific crosses, GISH verification of F_1_ chromosome constituents, and an examination of the microsporogenesis of the wild accession that was most effective in producing triploid F_1_ progenies, N102.

## Materials and Methods

### Plant materials

The wild species used in this study included 10 diploid species (2*n* = 2*x* = 34); *H. nuttallii* T. & G., *H. maximiliani* Schr., *H. mollis* Lam., *H. silphioides* Nutt., *H. divaricatus* L., *H. salicifolius* Dietr., *H. giganteus* L., *H. grosseserratus* Mar, *H. laciniatus* A. Gray, and *H. pumilus* Nutt; one tetraploid species (2*n* = 4*x* = 68) *H. hirsutus* Raf.; and two hexaploid species (2*n* = 6*x* = 102) *H. ciliaris* DC., and *H. resinosus* Small (Table 1). The cultivated sunflower parents included nuclear male-sterile (NMS), cytoplasmic male-sterile (CMS), and male-fertile (MF) maintainer and restoration lines; NMS HA 89-552, NMS P21, CMS HA 821, HA 89, P21, HA 821, HA 410, and RHA 274. All were publicly released by USDA. HA 89, HA 821, and HA 410 are oilseed maintainer lines, and RHA 274 is an oilseed restorer line. NMS HA 89-552 is an induced NMS mutant of inbred maintainer line HA 89 (Jan and Rutger 1988), and NMS P21 is a NMS mutant selected from open-pollinated variety Peredovik.

**Table 1:** Wild *Helianthus* species used in the study

Accession No.	Plant ID	Species	Ploidy	Collection Place	Year
102	N102	*H. nuttallii*	2*x*	Morden, MB, Canada	2003
314	N314	*H. nuttallii*	2*x*	Morden, MB, Canada	2005
622	N622	*H. nuttallii*	2*x*	Morden, MB, Canada	2001
PI 650025	NUT-RYD-2	*H. nuttallii*	2*x*	Kindred, ND	1993
1116	N1116	*H. nuttallii*	2*x*	Morden, MB, Canada	2005
1113	M1113	*H. maximiliani*	2*x*	Morden, MB, Canada	2005
PI 435753	MOL-2	*H. mollis*	2*x*	Okmulgee, OK	1984
PI 650013	MOL A 3201	*H. mollis*	2*x*	Chadds Ford, PA	1984
Ames 30356	G10/1163	*H. silphioides*	2*x*	Brandsville, MO	2009
PI 503209	G11/1301-10	*H. divaricatus*	2*x*	New Castle, VA	1985
PI 503216	G11/1320-39	*H. divaricatus*	2*x*	Kingston, NY	1985
Ames 30340	G10/1120-30	*H. salicifolius*	2*x*	Garnett, KS	2009
Ames 30348	G10/1131-37	*H. salicifolius*	2*x*	Ponca City, OK	2009
PI 547177	G07/15	*H*. *giganteus*	2*x*	Odanah, WI	1989
PI 613793	G07/25-27	*H*. *grosseserratus*	2*x*	Onawa, IA	1999
PI 547174	G10/1138-46	*H. hirsutus*	4*x*	Beecher City, IL	1989
PI 435648	CIL29-3	*H. ciliaris*	6*x*	Adrian, TX	1976
PI 650079	RES28382	*H. resinosus*	6*x*	Statesville, AL	2006
PI 650082	RES28386	*H. resinosus*	6*x*	Summit, MS	2006
PI 435707	LAC28-2	*H. laciniatus*	2*x*	San Lorenzo, TX	1977
405	N405	*H. nuttallii*	2*x*	Morden, MB, Canada	2005
187	N817	*H. nuttallii*	2*x*	Morden, MB, Canada	2005
1408	N1408	*H. nuttallii*	2*x*	Morden, MB, Canada	2005
902	N902	*H. nuttallii*	2*x*	Morden, MB, Canada	2005
903	N903	*H. nuttallii*	2*x*	Morden, MB, Canada	2005
412	N412	*H. nuttallii*	2*x*	Morden, MB, Canada	2005
PI 435860	PUM24-1	*H. pumilus*	2*x*	Boulder, CO	1977
PI 435860	PUM24B	*H. pumilus*	2*x*	Boulder, CO	1977
424	N424	*H. nuttallii*	2*x*	Morden, MB, Canada	2005
609	M609	*H. maximiliani*	2*x*	Morden, MB, Canada	2005
1008	N1008	*H. nuttallii*	2*x*	Morden, MB, Canada	2005
1324	N1324	*H. nuttallii*	2*x*	Morden, MB, Canada	2005
214	M214	*H. maximiliani*	2*x*	Morden, MB, Canada	2005
513	M513	*H. maximiliani*	2*x*	Morden, MB, Canada	2005
1018	M1018	*H. maximiliani*	2*x*	Morden, MB, Canada	2001
1314	M1314	*H. maximiliani*	2*x*	Morden, MB, Canada	2005
1323	M1323	*H. maximiliani*	2*x*	Morden, MB, Canada	2005
1418	M1418	*H. maximiliani*	2*x*	Morden, MB, Canada	2005

ID, identifier.

### Interspecific crossing and embryo rescue

Interspecific crosses of 15 accessions of *H. nuttallii* and *H. maximiliani* with cultivated sunflower were made in the greenhouse in 2006–2013 with the primary goal of introgressing *Sclerotinia* resistance (Table 2). The expanded reciprocal crosses between *H. nuttallii* N102 and other cultivated lines were made in the greenhouse in 2014 (Table 3). The reciprocal crosses of N102 with additional wild species accessions were made in the greenhouse in 2014 using N102 as male or female parents (Table 4 and Table 5). The crosses between perennial *H. divaricatus*, *H. salicifolius*, *H*. *giganteus*, *H*. *grosseserratus*, and *H. hirsutus*, and cultivated sunflower lines NMS HA 89-552 and HA 410, were made in the greenhouse in 2007–2012 (Table 6). MF female parents were emasculated for 3–4 d before pollination, with embryos of all the crosses rescued 6–8 d after pollination. The apical meristems of the F_1_ progeny seedlings were treated with 0.15% colchicine at pH = 5.4 for 5 hr in the dark for chromosome doubling following Jan and Chandler (1989).

**Table 2:** Number of diploid and triploid F_1_ progenies from *H. nuttallii* (N) and *H. maximiliani* (M) crossed with cultivated sunflower line NMS HA 89-552

Crosses	2*x* (2*n* = 34)	3*x* (2*n* = 51)	Triploids %	Total
NMS HA 89-552/N102	1	28	96.55	29
NMS HA 89-552/N314	0	2	100.00	2
NMS HA 89-552/N412	0	1	100.00	1
NMS HA 89-552/N1324	4	10	71.43	14
NMS HA 89-552/M1113	2	7	77.78	9
NMS HA 89-552/M1323	2	1	33.33	3
NMS HA 89-552/M1418	19	2	9.52	21
Subtotal	28	51	64.56	79
NMS HA 89-552/N1008	5	0	0.00	5
NMS HA 89-552/M1018	2	0	0.00	2
NMS HA 89-552/M513	1	0	0.00	1
NMS HA 89-552/M1314	5	0	0.00	5
NMS HA 89-552/M214	5	0	0.00	5
NMS HA 89-552/N405	2	0	0.00	2
NMS HA 89-552/N817	1	0	0.00	1
NMS HA 89-552/N1408	5	0	0.00	5
Subtotal	26	0	0.00	26
Total	54	51	48.57	105

NMS, nuclear male-sterile.

**Table 3:** Numbers of diploid or triploid F_1_ progenies from the crosses between *H. nuttallii* acc. 102 (N102) and cultivated sunflower lines

Crosses	Floret Number	2*x* (2*n* = 34)	3*x* (2*n* = 51)	Triploids %	Total Plants with 2*n*	Success %*^a^*
NMS HA 89-552/N102	11,830	4	94	95.92	98	0.83
NMS P21/N102	8,450	0	22	100.00	22	0.26
CMS HA 821/N102	7,750	0	5	100.00	5	0.06
Subtotal	28,030	4	121	96.80	125	0.45
HA 89/N102	11,560	9	28	75.68	37	0.32
RHA 274/N102	9,150	0	16	100.00	16	0.17
HA 821/N102	5,900	0	2	100.00	2	0.03
Subtotal	26,610	9	46	83.64	55	0.21
Total	54,640	13	167	92.78	180	0.33
N102/HA 89	8,140	17	0	0.00	17	0.21
N102/P21	2,060	28	0	0.00	28	1.36
N102/HA 821	5,020	7	0	0.00	7	0.00
N102/HA 410	8,090	18	0	0.00	18	0.22
Total	23,310	70	0	0.00	70	0.30

NMS, nuclear male-sterile; CMS, cytoplasmic male-sterile.

a Success % = total plants with 2*n* / floret number × 100.

**Table 4:** Mitotic chromosome numbers of F_1_ progenies from the crosses of nine wild *Helianthus* accessions × *H. nuttallii* N102

Crosses	Floret Number	2*n* = 33	2*n* = 34	2*n* = 51	2*n* = 68	2*n* = 85	Unexpected Polyploids %	Total Plants with 2*n*	Success %*^a^*
N314/N102	440	0	133	0			0.00	133	30.23
N622/N102	785	0	111	1			0.89	112	14.27
PI 650025/N102	595	0	85	1			1.16	86	14.45
M1113/N102	670	3	119	0			0.00	122	18.21
PI 435753/N102	4,585	0	111	0			0.00	111	2.42
N1116/N102	565	0	9	0			0.00	9	1.59
Ames 30356/N102	540	0	88	1			1.12	89	16.48
Total	8,180	3	656	3			0.45	662	8.09
PI 547174/N102	3,070			86	2		2.27	88	2.87
PI 435648/N102	1,520				32	1	3.03	33	2.17
Overall	12,770	3	656	89	34	1	0.77	783	6.13

a Success % = total plants with 2*n* / floret number × 100.

**Table 5:** Mitotic chromosome numbers of F_1_ progenies from the crosses of *H. nuttallii* N102 × seven wild *Helianthus* accessions, with N314/M1113 as a control

Crosses	Floret Number	2*n* = 32	2*n* = 33	2*n* = 34	2*n* = 36	2*n* = 51	2*n* = 68	2*n* = 85	Normal Plants %	Total Plants with 2*n*	Success %*^a^*
N102/N314	290	1	2	149	2	0			96.75	154	53.10
N102/N622	450			20		0			100.00	20	4.44
N102/PI 650025	150			34		0			100.00	34	22.67
N102/M1113	310			127		0			100.00	127	40.97
N102/PI 435753	1050		1	42		0			97.67	43	4.10
Total	2250	1	3	372	2	0			98.41	378	16.80
N102/PI 547174	980					8	0		100.00	8	0.82
N102/PI 435648	190						3	0	100.00	3	1.58
Total	1170					8	3	0	100.00	11	0.94
Overall	3420	1	3	372	2	8	3	0	98.46	389	11.37
N314/M1113	340			112		0				112	32.94

a Success % = total plants with 2*n* / floret number × 100.

**Table 6:** Mitotic chromosome numbers of F_1_ progenies from the crosses between cultivated sunflower and five other *Helianthus* species

Crosses	2*n* = 34	2*n* = 49	2*n* = 51	Abnormal Plants %	Total Plants with 2*n*
NMS HA 89-552/*H. divaricatus*	30	1	1	6.25	32
NMS HA 89-552/*H. salicifolius*	5			0.00	5
NMS HA 89-552/*H. giganteus*	7			0.00	7
NMS HA 89-552/*H. grosseserratus*	22			0.00	22
NMS HA 89-552/*H. hirsutus*			162	0.00	162
Total	64	1	163	0.88	228
* H. divaricatus*/HA 410	9			0.00	9
* H. salicifolius*/HA 410	26			0.00	26
* H. hirsutus*/HA 410			51	0.00	51
Total	35		51	0.00	86
Overall	99	1	214	0.64	314

NMS, nuclear male-sterile.

### Mitotic chromosome counts and GISH

Chromosome numbers in root tip cells were determined for individual plants using the standard Feulgen staining method. Chromosome squashes were made following the method of Liu *et al.* (2007) with minor modifications. F_1_ plants with 2*n* = 2*x* = 34 and 2*n* = 3*x* = 51 derived from different crosses were used for GISH analysis, according to Liu *et al.* (2013). Briefly, the root tips were digested at 37° for 2.5 hr in an enzyme mixture consisting of 2% cellulase (Sigma, St. Louis, MO) and 24% pectinase (Sigma) in 10 mM sodium citrate buffer (4 mM citric acid and 6 mM sodium citrate). The treated root tips were squashed in 45% acetic acid. Cover slips were removed from the slides after being frozen over liquid nitrogen for 5 min.

Genomic DNA of wild *Helianthus* species was used as a probe after being sheared in boiling water for 10 min and labeled with digoxingenin-11-dUTP using the nick translation method according to the manufacturer’s instructions (Roche Applied Science, Nutley, NJ). Genomic DNA of HA 89 was used as a blocking DNA after shearing in boiling water for 20 min and placed on ice for 5 min, with the ratio of blocking DNA to probe DNA of 50:1. Probe detection, image capture, and analysis followed Liu *et al.* (2013).

### Meiotic and mitotic division and pollen fertility analysis

Heads used for meiosis were collected and fixed in Carnoy’s fixative with 95% ethanol: chloroform: glacial acetic acid (6:3:1) at 4° for 24 hr, then rinsed several times with 70% ethanol and stored in 70% ethanol at 4° for analysis. The anthers at the appropriate developmental stage were placed on slides; pollen mother cells were squeezed out of the anther tissue and stained with 1% aceto-carmine. The pollen mother cells at different stages were analyzed using an Axioplan2 Imaging microscope (Zeiss, Germany). The pollen from undehisced anthers and open florets were stained with 1% aceto-carmine for pollen mitotic analysis at different stages of development.

The pollen fertility of N102, M1113, and 20 other perennial *Helianthus* accessions, cultivated HA 89, and the F_1_ plants was determined by pollen stainability using an Axioplan2 Imaging microscope. Pollen staining followed Alexander’s method (Alexander 1969). About 20 fields with a total of ∼1500–2000 pollen grains were observed for the *Helianthus* accessions and HA 89. Pollen grains were divided into four categories: large fertile (LF), large sterile (LS), small fertile (SF), and small sterile (SS). The SF pollen had a reduced chromosome number. For the F_1_s, five fields with a total of 300–500 pollen grains were analyzed. The percentage of each category was used for analysis. The pollen diameter was measured using Axiovision 4.8 software after the images were captured by a charge-coupled device camera (Zeiss AxioCam HRM).

### DNA extraction and PCR analysis

Genomic DNA was extracted according to the protocol of the QIAGEN DNAeasy 96 Plant Kit (QIAGEN, Valencia, CA). To identify the F_1_ plants (2*n* = 4*x* = 68) derived from *H. hirsutus* and *H. nuttallii* N102, six selected SSR markers mapped to the sunflower linkage groups from the Compositae database (http://compositdb.ucdavis.edu) were used for polymorphism screening between the parents, and the polymorphic primers were used for F_1_ genotyping. The PCR amplification and genotyping followed Liu *et al.* (2012).

### Backcrossing of diploid F_1_s and the production of amphiploids and aneuploids

Diploid F_1_s of NMS HA 89-552 crossed with *H. nuttallii* N102, N1008, and N1323, and *H. maximiliani* M1018, M1113, M1314, M1323, and M1418, were pollinated by cultivated sunflower HA 441 in 2006. The chromosomally-doubled heads of diploid F_1_s derived from *H. nuttallii* N412 and N1324, and *H. maximiliani* M1113, M1314, and M1323, were crossed with the triploid F_1_s derived from *H. nuttallii* N102 and N1324, and *H. maximiliani* M1113 and M1323 within the same species in 2006. The triploids from *H. nuttallii* N102, N314, and N1324 and *H. maximiliani* M1018, M1113, M1323, and M1418 were also used as the female parents crossed with HA 441 in 2006.

### Data availability

The authors state that all data necessary for confirming the conclusions presented in the article are represented fully within the article.

## Results

### Triploid hybrids obtained from diploid cultivated sunflower pollinated by diploid wild perennial species

**Figure 1:**
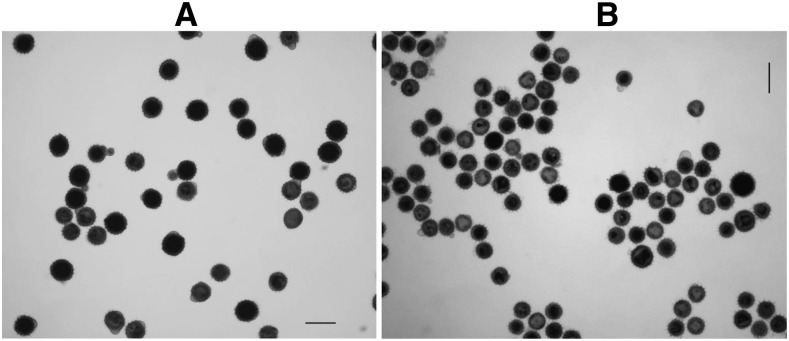
Pollen stainability of F_1_s with 2*n* = 3*x* = 51 (A) and 2*n* = 2*x* = 34 (B) derived from the cross of nuclear male-sterile (NMS) HA 89-552/*H. nuttallii* N102. The dark or black pollen grains are fertile, and the light or gray ones are sterile. Bars = 50 μm.

**Figure 2:**
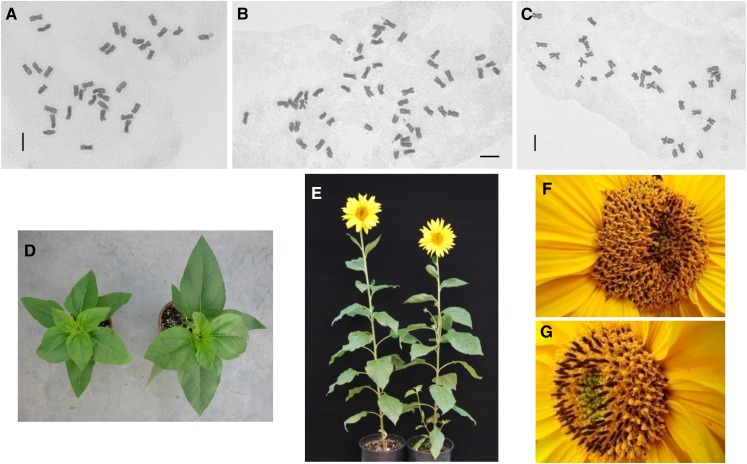
Chromosome squashes of diploid *H. nuttallii* N102 (A), triploid F_1_ (2*n* = 3*x* = 51) (B), and diploid F_1_ (2*n* = 2*x* = 34) (C) derived from the cross of nuclear male-sterile (NMS) HA 89-552/N102. Seedlings (D) of diploid (left) and triploid F_1_s (right), and adult plants (E) of diploid (left) and triploid F_1_s (right), flowering capitula of diploid (F) and triploid F_1_s (G), respectively. Bars = 5 μm.

*H. nuttallii* N102, N314, N405, N412, N817, N1008, N1324, and N1408, and *H. maximiliani* M214, M513, M1018, M1113, M1314, M1323, and M1418 (2*n* = 2*x* = 34) were crossed to cultivated NMS HA 89-552 (2*n* = 2*x* = 34) with the aim of producing amphiploids for improving the backcross seed set of the F_1_s (Table 2). The embryos were rescued and the F_1_ progeny seedlings were treated with colchicine. Then, the pollen stainability of 10 heads of each F_1_ plant was examined. LF pollen was predominant on heads of 28 of the 29 F_1_s derived from NMS HA 89-552/N102 (96.55%, Figure 1). This nearly 100% chromosome doubling is in contrast to the small number normally observed for colchicine-treated F_1_s in our prior experiments. In addition, all the heads of seven out of nine F_1_s derived from the cross of NMS HA 89-552/*H. maximiliani* M1113 also had a high percentage of LF pollen. High frequencies of large pollen grains were also observed in some F_1_s derived from other materials tested without colchicine treatment, which suggested that the high percentage of LF pollen was not the result of colchicine treatment. The subsequent mitotic chromosome analysis for the F_1_ progenies demonstrated that 51 plants had 2*n* = 3*x* = 51 (48.57%) and 54 plants had 2*n* = 2*x* = 34 (51.43%). Triploid F_1_s were detected from the crosses involving seven *H. nuttallii* and *H. maximiliani* accessions, with a high frequency of 64.56% (plant number *n* = 79), but not detected for 26 F_1_ plants from eight other accessions (Table 2). Among the accessions, *H. nuttallii* N102 and *H. maximiliani* M1113 produced the highest frequency of triploid F_1_s. Since the corresponding wild accessions were confirmed to be diploid with 2*n* = 2*x* = 34 (Figure 2A for N102), we used GISH analysis to confirm the chromosome constituents of the 2*n* = 3*x* = 51 F_1_s.

### GISH analyses of F_1_ hybrids derived from interspecific crosses involving H. nuttallii and H. maximiliani

**Figure 3:**
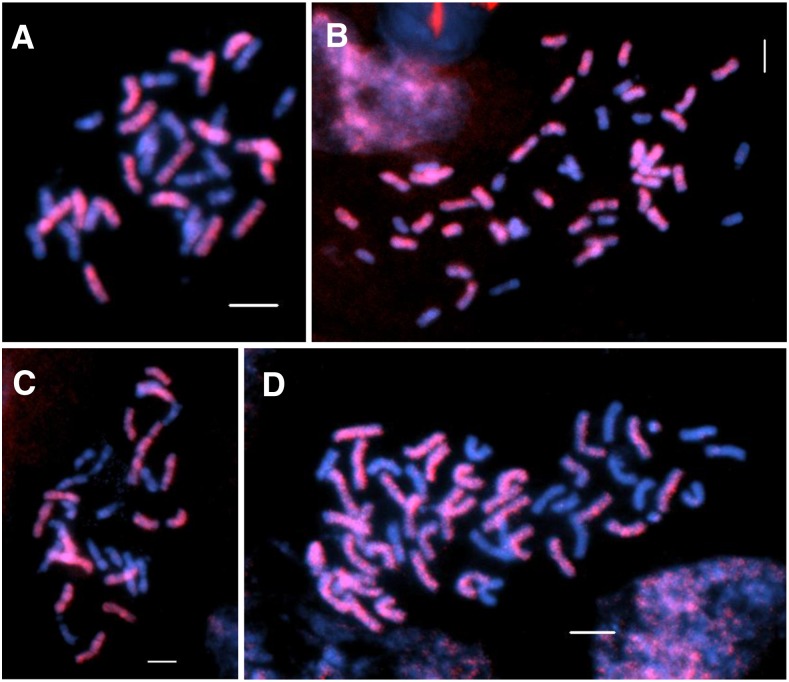
GISH analyses of an F_1_ diploid NMS HA 89-552/M214 (A), triploid NMS HA 89-552/*H. nuttallii* N102 (B), diploid NMS HA 89-552/N817 (C), and triploid NMS HA 89-552/*H. maximiliani* M1113 (D). The genomic DNA of *H. nuttallii* N102 and *H. maximiliani* M1113 were labeled with dig-11-dUTP and detected by anti-dig-rhodamine (red), the chromosomes were counterstained by DAPI (blue). Bars = 5 μm. DAPI, 4’,6-diamidino-2-phenylindole; dig, digoxigenin; dUTP, 2’-deoxyuridine 5’-triphosphate; GISH, genomic *in situ* hybridization; NMS, nuclear male-sterile.

Using the genomic DNA of N102 and M1113 as the probe DNA, respectively, and HA 89 as a blocking DNA, the F_1_ individuals with 2*n* = 3*x* = 51 and 2*n* = 2*x* = 34 were analyzed by the GISH technique for genome compositions (Figure 3). The results clearly showed that, among the 51 chromosomes of the triploid F_1_s, 34 chromosomes had signals of the two wild *Helianthus* species (red) and the remaining 17 chromosomes had no probe signals (blue) (Figure 3, B and D). In comparison, only 17 chromosomes were stained by the two probes in the diploid F_1_ individuals with 2*n* = 2*x* = 34 (Figure 3, A and C). Therefore, GISH analysis indicated that the triploids arose from an excess paternal genome contribution.

### Triploid F_1_ hybrids not related to the male sterility of female parents

To determine if the abnormally high frequency of triploids was due to the use of the male-sterile line as the female parent in the interspecific hybridizations, N102 pollen was applied to two NMS lines (NMS HA 89-552 and NMS P21), one CMS line (CMS HA 821), and three MF lines (HA 89, RHA 274, and HA 821) (Table 3). A total of 180 F_1_ plants were obtained from 54,640 florets (0.33%) using embryo rescue. For NMS HA 89-552/N102, 95.92% of F_1_ plants were triploids (Figure 2B) (*n* = 98), with only four normal diploids (Figure 2C). The triploid and diploid plants looked similar, except that the triploids appeared more vigorous at the seedling stage, were more branched as adults, produced higher amounts of pollen, and had higher pollen stainability (Figure 2, D–G).

In addition, all 27 F_1_s derived from NMS P21/N102 and CMS HA 821/N102 were triploids. The overall percentage of triploids obtained from the MS female parents × N102 was 96.80% (*n* = 125). For the MF female parents × N102, a lower percentage of triploids was obtained from the cross HA 89/N102 (75.68%, *n* = 37), and all 18F_1_s derived from RHA 274/N102 and HA 821/N102 were triploids. The overall number of triploids obtained from the MF female parents × N102 was 83.64% (*n* = 55). In summary, 92.78% of the F_1_s derived from the both MF and MS female parents were triploids (*n* = 180), and triploid F_1_ hybrid production was not related to the male sterility of female parents.

### Only normal ploidy levels were observed when N102 was pollinated with cultivated sunflower

To examine whether abnormal triploids would be obtained from crosses when N102 was used as the female parent, N102 was pollinated with pollen from four cultivated sunflower lines (HA 89, P21, HA 821, and HA 410). Seventy F_1_ plants from 23,310 florets were obtained for mitotic chromosome counts after embryo rescue (0.30%). However, all the F_1_s had 2*n* = 2*x* = 34, which was in stark contrast to the results obtained when N102 was used as the male parent. The results suggested that the female megasporogenesis of N102 was normal, which led us to suspect the possible abnormal pollen meiosis, pollen mitotic division, pollen fertility, or fertilization process of N102.

### Pollen fertility and grain size of wild Helianthus accessions

**Figure 4:**
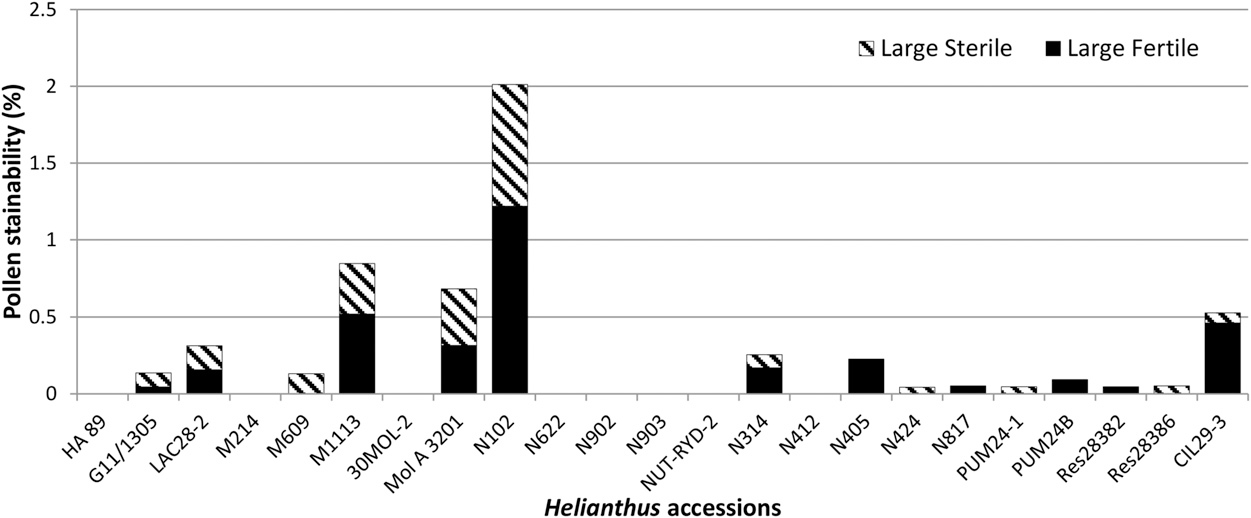
The total percentage of large fertile and large sterile pollen grains of 22 wild *Helianthus* accessions including *H. nuttallii* N102 and *H. maximiliani* M1113, with HA 89 as a control.

Pollen fertility was analyzed for 22 wild *Helianthus* accessions including N102 and M1113, with HA 89 as a control (Figure 4 and Supplemental Material, File S1, Table S1). For N102, 95.18% of the pollen was SF, with 1.22% LF, 0.79% LS, and 2.80% SS (*n* = 1640, Figure 5A). The total large pollen was 2.01%. The total of LF and LS grains for M1113 was 0.85%, slightly lower than that of N102, with 90.89% SF, 0.52% LF, 0.33% LS, and 8.26% SS (*n* = 1537). Nine other *Helianthus* accessions had low frequencies of LF grains (range = 0.05–0.46%), with or without LS grains. However, four other *Helianthus* accessions only contained a low percentage of LS grains (range = 0.04–0.13%). The average LF and LS grains for the 22 wild *Helianthus* accessions were 0.15 and 0.10%, respectively. In comparison, N102 and M1113 combined produce over 10 times more large pollen grains than the other *Helianthus* accessions. For comparison, HA 89 did not produce any large grains, with 95.98% SF and 4.02% SS (*n* = 1715, Figure 5B). In addition, the total percentage of sterile male gametes (LS + SS) in N102 and M1113 was 3.60 and 8.59%, respectively. In comparison, except for five accessions having very high male sterility (above 20%), the average percentage of sterile male gametes of the 15 other *Helianthus* accessions was 2.23% (Table S1). The high sterility of the five accessions was mainly caused by pollen size differentiation and partial staining of the pollen grains.

**Figure 5:**
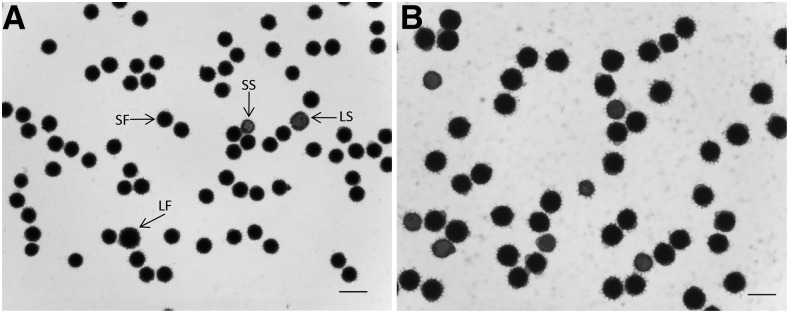
Pollen stainability of *H. nuttallii* N102 (A) and HA 89 (B). Bars = 50 μm. The large fertile (LF), large sterile (LS), small fertile (SF), and small sterile (SS) pollen grains are indicated by arrows in (A).

Measurements of normal and LF pollen grains indicated that the average diameter of normal and large pollen of N102 was 26.19 μm (*n* = 150, range = 23.99–28.23 μm) and 33.82 μm (*n* = 35, range = 31.42–35.74 μm), respectively (Figure 5A). The ratio of normal *vs.* “large” fertile pollen was 1:1.29 for N102. The preliminary analysis of the diameter of normal and “large” pollen for six other *Helianthus* accessions indicated that the average size ratio of normal *vs.* “large” pollen was 1:1.27 (range = 1:1.22–1:1.37). Therefore, the diameter of “large” fertile pollen was nearly 30% larger than that of normal fertile pollen for these *Helianthus* accessions.

### Meiotic and pollen mitotic analyses of N102

**Figure 6:**
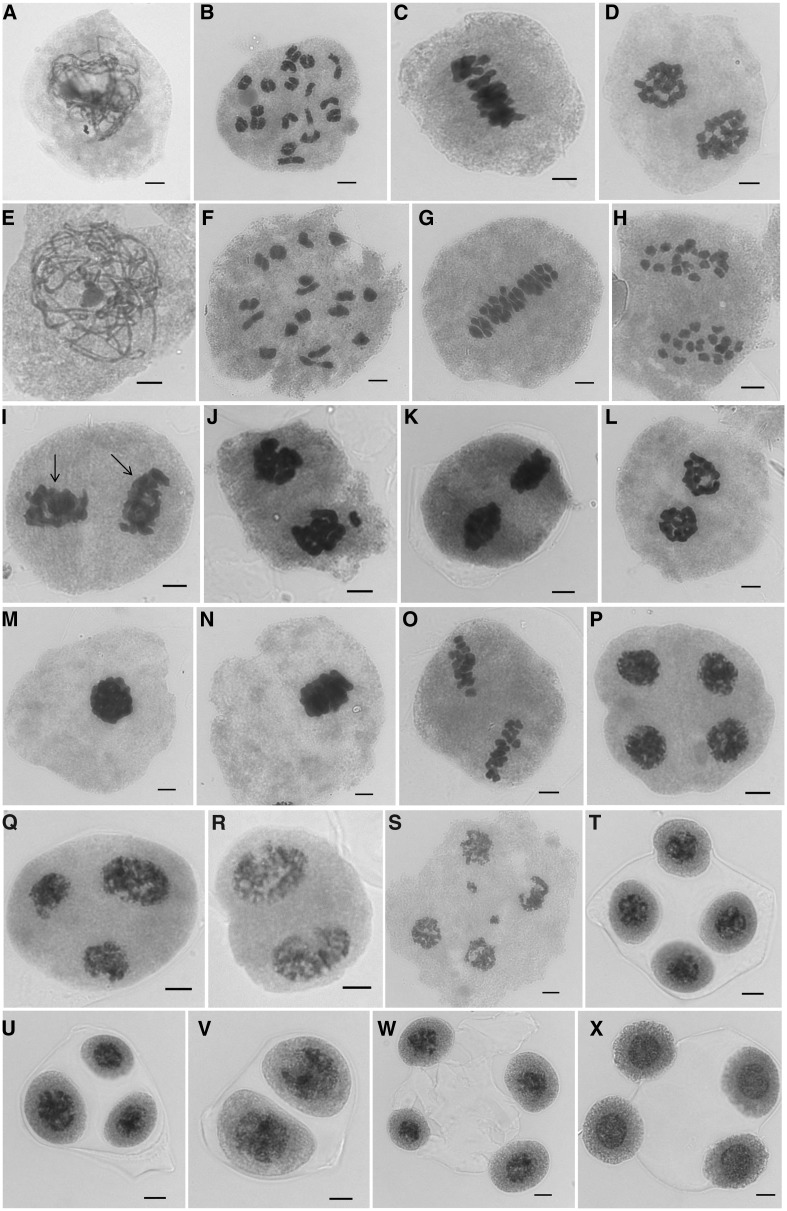
Meiotic analysis of *H. nuttallii* N102 and HA 89. (A–D) Meiosis I of N102. (E–H) Meiosis I of the cultivated line HA 89. (A and E) Pachytene. (B and F) Diakinesis. (C and G) Metaphase I. (D and H) Telophase I. (I–N) Meiosis II of N102. (I) Two well separated metaphase II plates, indicated by arrows. (J) Two well-separated metaphase II plates, with one lagging chromosome. (K and L) Two metaphase II plates at the same angle, which were spread paralleled. (M and N) Two metaphase II plates were squashed as one cluster. (O) Meiosis II of HA 89, showing two well-separated plates. (P–S) Telophase II of N102. (P) Cell with four normal nuclei. (Q) Cell with one large nucleus (two merged nuclei) and two normal nuclei. (R) Cell with two large nuclei. (S) Cell with four nuclei and two lagging chromosomes. (T–W) Tetrad stage of N102. (T) Normal tetrad. (U) Triad. (V) Dyad. (W) Unbalanced type at the tetrad stage. (X) Tetrad of HA 89. The meiosis I was normal for both materials. Abnormality was noticed at meiosis II for N102. Big nucleus resulted from the merging of two normal nucleuses were observed at telophase II. Low percentages of triads and dyads were noticed at meiosis II. Bars = 5 μm.

Meiosis in N102 progressed similarly to the cultivated line HA 89 (Figure 6, E–H) until the end of telophase I (Figure 6, A–D). No preduplication was observed before meiosis at interphase. At diakinesis, the 17 bivalents could be identified. At metaphase I, the bivalents were aligned on the metaphase plate, with a few cells having one, two, or even four chromosomes separated early. At telophase I, the homologous chromosomes were separated, with a few cells having one or two chromosomes lagging behind. HA 89 had a normal meiosis I, with no lagging chromosomes (Figure 6, E–H).

At metaphase II, two well-separated metaphase II plates were observed (indicated by arrows in Figure 6I), with 8.57% showing one or two lagging chromosomes (*n* = 210) (Figure 6J). However, some cells appeared to contain two plates at the same angle, which were spread parallel as two plates (Figure 6, K and L) or squashed as one cluster (Figure 6, M and N) (with the latter type excluded from calculation to avoid confusion with metaphase I), suggesting the possible existence of parallel spindles (compare Figure 6, K–N with Figure 6I). In comparison, HA 89 had two well-separated metaphase II plates (Figure 6O). At telophase II, 96.98% of the cells had four normal nuclei (Figure 6P), with 0.79% of cells containing one large nucleus (two merged nuclei) and two normal nuclei (Figure 6Q), 0.22% containing two large nuclei (Figure 6R), and 2.01% having other unbalanced meiotic products (such as three or four normal nuclei and one or two tiny nuclei, or with lagging chromosomes, Figure 6S) (*n* = 1392). In total, we observed 94.55% normal tetrads (four sets of 17 chromosomes, Figure 6T), 4.61% triads (two sets of 17 chromosomes and one set of 34, Figure 6U), 0.82% dyads (two sets of 34 chromosomes, Figure 6V), and 0.03% other unbalanced types at the tetrad stage (Figure 6W) (*n* = 3431). Large daughter cells with two nuclei were also observed. HA 89 had a normal meiosis II, with 100% tetrad being observed at the tetrad stage (Figure 6X) (*n* = 2490), except for a few cells having nuclei of slightly different sizes.

Correspondingly, 1.62% large microspores were observed in N102 (*n* = 1544). These results suggest that a low percentage of chromosome nonreduction for N102 occurred during meiosis II, especially at the telophase II and tetrad stages, leading to the different sizes of pollen grains. Therefore, pollen size could be used to infer ploidy level of pollen grains, with normal or small pollen size corresponding to reduced microspores, and “large” pollen corresponding to unreduced 2*n* microspores, respectively.

Pollen mitotic analysis indicated a normal process for N102 (Figure S1). Of the nearly 1400 mature pollen grains analyzed, all contained one vegetative nucleus and two spermatids, including the large pollen (1.07%). With only ∼1% unreduced pollen compared to 99% normal pollen in N102, differential fertilization favoring unreduced pollen certainly played an important role in the production of more triploids than the normal diploid F_1_ hybrids, when N102 was used as the pollen source.

### Rare abnormal chromosome numbers observed in the progenies derived from the crosses between N102 and wild perennial Helianthus species

To determine whether the triploids were obtained specifically from the interspecific crosses between N102 and cultivated sunflower, intraspecific and interspecific crosses were made between N102 and diploid *H. nuttallii* N314, N622, PI 650025, N1116, *H. maximiliani* M1113, *H. mollis* (PI 435753), and *H. silphioides* (Ames 30356); tetraploid *H. hirsutus* (PI 547174); and hexaploid *H. ciliaris* (PI 435648); with N102 as the male (Table 4) or female parent (Table 5). Noticeably, N314 and M1113 were also observed to produce triploids when they were crossed to NMS HA 89-552. A total of 662 F_1_ plants (8.09%) were obtained from 8180 florets from the crosses involving seven diploid perennials with N102 as the male parent after embryo rescue (success rate = 1.59–30.23%). However, only three F_1_ plants from the crosses involving *H. nuttallii* N622, PI 650025, and *H. silphioides* (Ames 30356) (Figure S2, A–F) were triploids (0.45%). The percentages of triploids from the three crosses were 0.89, 1.16, and 1.12%, respectively (Table 4).

For the cross *H. hirsutus* (PI 547174, 2*n* = 4*x* = 68)/N102, 88 F_1_ plants (2.87%) were obtained from 3070 florets (Table 4). Two tetraploid F_1_ plants (2.27%, 2*n* = 4*x* = 68) were detected, which were confirmed using SSR marker ORS505 from linkage group 5 of the sunflower SSR map (Figure S2G for 2*n* = 3*x* = 51, Figure S2H for 2*n* = 4*x* = 68, and Figure S3). For the cross *H. ciliaris* (PI 435648, 2*n* = 6*x* = 102)/N102, 33 F_1_ plants (2.17%) were obtained from 1520 florets. Except for one with 2*n* = 5*x* = 85 (3.03%), all F_1_ plants had 2*n* = 4*x* = 68 (Figure S2I for 2*n* = 4*x* = 68 and Figure S2J for 2*n* = 5*x* = 85). The overall percentage of abnormal F_1_ plants was 0.77% of the 783 progenies from the crosses of nine wild *Helianthus* accessions × N102. In addition, three plants had 2*n* = 2*x* − 1 = 33 (Figure S2, K and L) from the cross of M1113/N102 (2.46%, *n* = 122). In these crosses, the ratio of F_1_ hybrids appears to reflect the percentage of reduced and unreduced pollen of N102, and the lack of differential selection of reduced and unreduced pollen grains was obvious.

By comparison, of the F_1_s derived from the seven crosses with N102 as the female parent, no unexpected polyploid plants were obtained. Only six plants had abnormal chromosome numbers with 2*n* = 32, 33, or 36 (1.54%, *n* = 389), with five plants derived from N102/N314 and one from N102/*H. mollis* (PI 435753) (Figure S2, M–O and Table 5). The overall success rate for embryo rescue was 11.37%, with 16.34% for the five crosses involving diploid *Helianthus* perennials, and 0.82 and 1.58% for the crosses involving the tetraploid and hexaploid *Helianthus* perennials, respectively. In addition, all 112 F_1_s derived from N314/M1113 were normal diploids, *i.e.*, 2*n* = 2*x* = 34, with a success rate of 32.94%.

### Low frequency of abnormal chromosome numbers in progenies of interspecific crosses involving other perennial species

*H. salicifolius*, *H. divaricatus*, *H. hirsutus*, *H*. *giganteus*, and *H*. *grosseserratus* were also crossed with NMS HA 89-552 and HA 410, with the aim of transferring useful agronomic genes into cultivated sunflower. The F_1_ seedlings were obtained through embryo rescue, and mitotic chromosome counts taken (Table 6). Only one plant with 2*n* = 3*x* = 51 and one with 2*n* = 3*x* − 2 = 49 were detected (6.25%) in the 32 F_1_ progenies derived from the cross NMS HA 89-552/*H. divaricatus* (Figure S2, P–R). Additionally, the pollen stainability analysis indicated that one *H. divaricatus* accession had 0.05% LF and 0.09% LS pollen grains. The overall percentage of plants with abnormal chromosome numbers was 0.64% (*n* = 314). Compared to the > 75% of triploids obtained from the crosses with N102 as the male parent and cultivated sunflower as the female parent, the frequency of plants with abnormal chromosome numbers in these interspecific crosses was very low, likely corresponding to a low frequency of unreduced pollen in these species.

### Diploid and triploid progenies used to introgress genes from wild into cultivated sunflower by backcrossing and producing amphiploids/aneuploids

Backcrosses of triploid F_1_s of NMS HA 89-552/*H. nuttallii* N102, N1008, or N1323, and NMS HA 89-552/*H. maximiliani* M1018, M1113, M1314, M1323, or M1418 with HA 441 produced no seed, and the diploid F_1_s crossed with HA 441 had very low seed set. However, most of the backcross progenies of the diploid F_1_s with HA 441 as the pollen source had 2*n* = 2*x* = 34, with 37.14% of the progeny having 2*n* = 2*x* + 1 = 35 (*n* = 32). The average selfed seed set was 36.38% (range = 0.17–95%), and the backcrossed seed set was 18.84% (range = 2.00–60%). These results would be consistent with unbalanced chromosome pairing in the interspecific F_1_s. However, 41.38% of progeny had 20% or greater backcross seed set, demonstrating the practicality of utilizing these 2*n* = 2*x* = 34 hybrids to introgress genes from wild species into cultivated sunflower.

**Figure 7:**
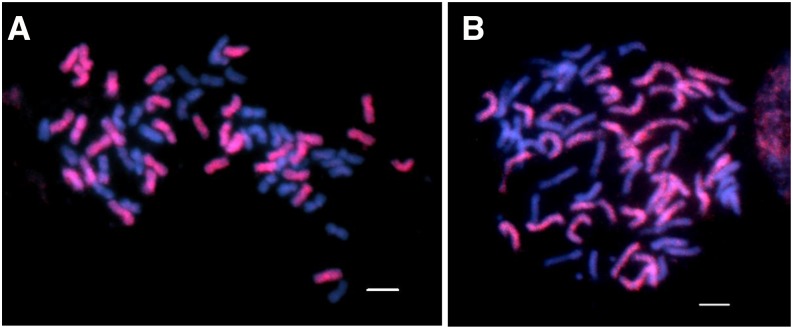
GISH analyses of two amphiploid individuals derived from NMS HA 89-552/*H. maximiliani*. (A) G06/783-3, 2*n* = 4*x* − 2 = 66. (B) G06/783-9, 2*n* = 4*x* − 1 = 67. The genomic DNA of *H. maximiliani* M1418 was labeled with dig-11-dUTP and detected by anti-dig-rhodamine (red), the chromosomes were counterstained by DAPI (blue). Bars = 5 μm. DAPI, 4’,6-diamidino-2-phenylindole; dig, digoxigenin; dUTP, 2’-deoxyuridine 5’-triphosphate; GISH, genomic *in situ* hybridization; NMS, nuclear male-sterile.

One tetraploid amphiploid with 2*n* = 67–69 was produced by crossing chromosomally doubled heads of colchicine-treated diploid F_1_ plants of NMS HA 89-552/N412 with the untreated pollen of triploid F_1_ plants of NMS HA 89-552/N102. Similarly, another tetraploid amphiploid with 2*n* = 66–68 was produced by crossing chromosomally doubled heads of colchicine-treated diploid F_1_ plants of NMS HA 89-552/M1323 with the untreated pollen of triploid NMS HA 89-552/M1113. Mitotic GISH analysis of two individuals of one amphiploid derived from NMS HA 89-552/M1323//NMS HA 89-552/M1113 indicated that about half of the chromosomes were from *H. maximiliani* with the others from cultivated sunflower (Figure 7). This is only possible when the unreduced female gametes were fertilized by balanced unreduced gametes having 2*n* = 2*x* = 34, with half from the wild parent and half from cultivated line. The seed set for sib-crosses and selfs of the *H. nuttallii* amphiploid were 47.19 and 50.47%, respectively. For the *H. maximiliani* amphiploid, the seed set for sib-crosses was 25%. Embryo rescue was not needed to produce seeds. Therefore, these amphiploids provide useful bridge materials for introgression of important agronomic traits into cultivated sunflower.

A series of aneuploids from crosses of the triploid F_1_s of NMS HA 89-552/*H. nuttallii* N102, N314, or N1324, and NMS HA 89-552/*H. maximiliani* M1018, M1113, M1323, or M1418 with HA 441 were obtained utilizing embryo rescue. Chromosome numbers of the BC_1_F_1_ progeny ranged from 2*n* = 37 to 51. However, the average seed set was very low (0.39%) with additional backcrosses with HA 441 due to the unbalanced chromosome numbers. These materials could be used for further backcrossing with the aim of reducing the chromosome number and improving fertility.

## Discussion

### Triploids provided evidence of abnormal polyploidization of sunflower

In almost all plant species, 2*n* gametes were frequently observed in interspecific or interploidy crosses, and could be triggered by abiotic and biotic stresses, such as heat and cold shocks, water stress, nutrition, and disease (Storme and Mason 2014; Mason and Pires 2015). It has been assumed that 2*n* gametes occur only rarely and the importance of 2*n* gametes in the origin of polyploids has been argued [Harlan and deWet 1975; Ramanna and Jacobsen 2003; reviewed by Dewitte *et al.* (2012)]. However, accumulating evidence suggests that unreduced gametes may have had a major role in the evolution of polyploids (Tayalé and Parisod 2013).

In this study, a high frequency of triploids was observed in interspecific crosses of two diploid perennial *Helianthus* species, specifically N102, pollinated to cultivated sunflower. Mitotic analysis of nearly 1800 progenies indicated that the frequency of triploid F_1_ progenies was 92.78% for the crosses of cultivated lines/N102, which was significantly higher than the 0.77% of unexpected polyploidy plants obtained from the crosses of other wild perennial *Helianthus* species/N102. No unexpected polyploids were observed in crosses of N102/cultivated sunflower, or N102/other perennial *Helianthus* species. By comparison, the percentages of progenies with abnormal chromosome numbers derived from five other perennial *Helianthus* species pollinated onto the cultivated NMS HA 89-552 was only 0.64%. A very low frequency of 2*n* pollen was observed in N102 (1.22%) and M1113 (0.52%). However, they still had the highest percentage of 2*n* pollen among the 22 wild *Helianthus* accessions studied, especially N102, but closely agree with the estimate of 2*n* gametes production in nonhybrid flowering plants (0.56%) (Ramsey and Schemske 1998). The results indicated that the female gametes of N102 are normal. The analysis of the pollen fertility of 22 wild *Helianthus* accessions and HA 89 suggested that the sterile male gametes normally exist in wild *Helianthus* species and cultivated sunflower, and that N102 and M1113 had a similar or higher male sterility than most of the other wild *Helianthus* accessions studied. On the other hand, the low frequency of 2*n* male gametes of N102 suggested that the high frequencies of triploids were caused by a higher compatibility of 2*n* gametes, relative to the reduced male gametes of N102, to the female cultivated sunflower. However, the difference between the 2*n* and *n* gametes was not as evident when N102 was pollinated to other perennial *Helianthus* species. In the latter case, low frequencies of unexpected polyploids were produced.

Therefore, the high frequency of triploid F_1_s derived from diploid × diploid crosses provided evidence of extreme abnormal polyploidization of sunflower. Since the cross-incompatibility between perennial wild *Helianthus* and cultivated lines is high, these triploids are likely the results of the low frequency of 2*n* male gametes under specific crossing pressure, *i.e.*, due to the dosage balance of factors related to recognition and rejection of foreign pollen during fertilization (Mason and Pires 2015). This unique triploid F_1_ production will expedite our understanding of sunflower evolution and speciation, and the effect of unreduced gametes on crossing compatibility between *Helianthus* species.

### Triploids and amphiploids are valuable for genetic study and plant breeding

The semifertile triploid F_1_ progenies derived from interspecific crosses of cultivated sunflower pollinated by the two wild *Helianthus* species have been used for developing amphiploids and aneuploids, and for interspecific gene transfer. Therefore, these triploids could act as an important bridge during the evolution and speciation of sunflower. Furthermore, these triploids could be a valuable tool for producing alien addition lines or monosomic lines and chromosome number tracking to reveal the effects of individual chromosomes. Under specific circumstances, alien addition lines will facilitate molecular gene mapping when the gene is linked with the alien chromosome, similar to the strategy for the mapping of the male fertility restoration gene *Rf_6_* (Liu *et al.* 2013).

Amphiploids with improved seed set can be maintained by sib-pollination or produce backcross seed without embryo rescue, and reduce the effort required to introduce the genetic diversity in the perennial species into cultivated sunflower. Amphiploids are a useful “bridge” for transferring important agronomic traits into cultivated sunflower. For example, a broomrape resistance gene for race F in Spain has been successfully transferred from a wild *Helianthus* species into cultivated sunflower using amphiploids (Pérez-Vich *et al.* 2002), and two male fertility restoration genes, *Rf_4_* and *Rf_6_*, were identified in amphiploids and introgressed into cultivated sunflower (Feng and Jan 2008; Liu *et al.* 2013). Amphiploids could also enable crosses between species of different ploidy levels, further increasing the genetic diversity of cultivated sunflower.

### Reasons for the unexpected triploids derived from the two wild Helianthus species

Triploids can form by fusion of a 2*n* gamete with a normal haploid gamete or by polyspermy (Ravi *et al.* 2008). Unreduced gametes most commonly arise through meiotic defects, including the omission of the first or second meiotic division, abnormal spindle morphology in the second division, or disturbed cytokinesis (Brownfield and Köhler 2011). Several plant mutants producing triploid offsprings have been molecularly identified and characterized, such as *DYAD/SWITCH1*, *RBR*, and *AGO104* (Ravi *et al.* 2008; Chen *et al.* 2009; Singh *et al.* 2011). A high frequency of triploids (80%) has been associated with the *dyad* allele of *SWI1* of *Arabidopsis*, which is expressed as an absence of meiosis in the female gamete formation caused by mutation of *DYAD/SWITCH1* (Ravi *et al.* 2008). The *Arabidopsis* triploid rbr/+ offsprings presumably resulted from the fusion of a haploid egg with a diploid rbr sperm that originated from a postmeiotic genome duplication event (Chen *et al.* 2009). A maize mutant, *Dnr 4* at locus *AGO104*, also results in the formation of functional unreduced gametes because of the defects in chromatin condensation during meiosis (> 80% of the meiocytes at metaphase I) (Singh *et al.* 2011). Triploid progeny could also be the result of 2*x* × 2*x* sexual hybridization in *Citrus* as a consequence of the formation of unreduced gametes at a low frequency (Aleza *et al.* 2010).

In this study, the low percentage of chromosome nonreduction in N102 was caused by meiosis II nonreduction. At the telophase II stage, we observed cells containing three spores (two haploid spores and one diploid one) and two diploid spores. We also observed a low percentage of triads and dyads at the tetrad stage. It appeared to be the consequence of tripolar or paralleled spindles at meiosis II for chromosome nonreduction, which may be caused by defects in the interzonal microtubule array (d’Erfurth *et al.* 2008; Storme and Geelen 2013). Since the triploid production using *H. nuttallii* N102 pollen has been observed over a long period of time, and under a range of environmental conditions, it is likely that the consistency of the frequent unreduced male gametes that result in the relatively high frequency of F_1_ triploids is due to unique genetic alterations.

### Future study

The molecular mechanism for the occurrence of the high frequency of triploid production in the interspecific crosses involving the two diploid wild *Helianthus* species and cultivated sunflower lines remains to be further evaluated. Among the questions awaiting answers are: (1) the relative competitiveness of large and small pollen on fertilization, using diploid and tetraploid P21 pollens, and their mixed pollen to N102, or using N102 and chromosome-doubled N102 to cultivated sunflower; (2) examine pollen germination on the stigmas of NMS HA 89-552 and the fertilization process using confocal scanning microscopy; and (3) study the genetics causing the high frequency of unreduced male gametes.

## Supplementary Material

Supplemental material is available online at www.g3journal.org/lookup/suppl/doi:10.1534/g3.116.036327/-/DC1.

## References

[bib1] Alexander M. P., 1969 Differential staining of aborted and nonaborted pollen. Stain Technol. 44: 117–122. 4181665 10.3109/10520296909063335

[bib2] Aleza P. Juárez J. Cuenca J. Ollitrault P. Navarro L., 2010 Recovery of *Citrus* triploid hybrids by embryo rescue and flow cytometry from 2x × 2x sexual hybridization and its application to extensive breeding programs. Plant Cell Rep. 29: 1023–1034. 20607244 10.1007/s00299-010-0888-7

[bib3] Brownfield L. Köhler C., 2011 Unreduced gamete formation in plants: mechanisms and prospects. J. Exp. Bot. 62: 1659–1668. 21109579 10.1093/jxb/erq371

[bib4] Chandler J. M. Beard J. M., 1983 Embryo culture of *Helianthus* hybrids. Crop Sci. 23: 1004–1007.

[bib5] Chen Z. Hafidh S. Poh S. H. Twell D. Berger F., 2009 Proliferation and cell fate establishment during *Arabidopsis* male gametogenesis depends on the Retinoblastoma protein. Proc. Natl. Acad. Sci. USA 106: 7257–7262. 19359496 10.1073/pnas.0810992106 PMC2678419

[bib6] d’Erfurth I. Jolivet S. Froger N. Catrice O. Novatchkova M., 2008 Mutations in *AtPS1* (*Arabidopsis thaliana parallel spindle 1*) lead to the production of diploid pollen grains. PLoS Genet. 4: e1000274. 19043546 10.1371/journal.pgen.1000274 PMC2581889

[bib7] Dewitte A. Van Laere K. Van Huylenbroeck J., 2012 Use of 2n gametes in plant breeding, pp. 59–86 in Plant Breeding, edited by Abdurakhmonov I. Y. InTech, Rijeka, Croatia.

[bib8] Feng J. Jan C. C., 2008 Introgression and molecular tagging of *Rf_4_*, a new male fertility restoration gene from wild sunflower *Helianthus maximiliani* L. Theor. Appl. Genet. 117: 241–249. 18437344 10.1007/s00122-008-0769-4

[bib9] Harlan J. R. deWet J. M. J., 1975 On Ö. Winge and a prayer: the origins of polyploidy. Bot. Rev. 41: 361–390.

[bib10] Henry I. M. Dilkes B. P. Young K. Watson B. Wu H., 2005 Aneuploidy and genetic variation in the *Arabidopsis thaliana* triploid response. Genetics 170: 1979–1988. 15944363 10.1534/genetics.104.037788 PMC1449780

[bib11] Jaillon O. Aury J. M. Noel B. Policriti A. Clepet C., 2007 The grapevine genome sequence suggests ancestral hexaploidization in major angiosperm phyla. Nature 449: 463–467. 17721507 10.1038/nature06148

[bib12] Jan C. C., 1997 Cytology and interspecific hybridization, pp. 497–558 in Sunflower Technology and Production, edited by Schneiter A. A. ASA, CSSA, SSSA, Madison, WI.

[bib13] Jan C. C. Rutger J. N., 1988 Mitomycin C- and streptomycin-induced male sterility in cultivated sunflower. Crop Sci. 28: 792–795.

[bib14] Jan C. C. Chandler J. M., 1989 Sunflower interspecific hybrids and amphiploids of *Helianthus annuus* × *H. bolanderi*. Crop Sci. 29: 643–646.

[bib15] Jan C. C. Feng J. Seiler G. J. Rashid K. Y., 2007 Development of *Sclerotinia* head rot resistant germplasm utilizing *H. maximiliani* and *H. nuttallii*. 29th Sunflower Research Workshop, January 10-11, 2007, Fargo, ND.

[bib16] Jiao Y. Wickett N. J. Ayyampalayam S. Chanderbali A. S. Landherr L., 2011 Ancestral polyploidy in seed plants and angiosperms. Nature 473: 97–100. 21478875 10.1038/nature09916

[bib17] Liu Z. Li D. Y. Zhang X. Y., 2007 Genetic relationships among five basic genomes St, E, A, B and D in Triticeae revealed by genomic southern and *in situ* hybridization. J. Integr. Plant Biol. 49: 1080–1086.

[bib18] Liu Z. Mulpuri S. Feng J. Vick B. A. Jan C. C., 2012 Molecular mapping of the *Rf_3_* fertility restoration gene to facilitate its utilization in breeding confection sunflower. Mol. Breed. 29: 275–284.

[bib19] Liu Z. Wang D. Feng J. Seiler G. J. Cai X., 2013 Diversifying sunflower germplasm by integration and mapping of a novel male fertility restoration gene. Genetics 193: 727–737. 23307903 10.1534/genetics.112.146092 PMC3583994

[bib20] Mason A. S. Pires J. C., 2015 Unreduced gametes: meiotic mishap or evolutionary mechanism? Trends Genet. 31: 5–10. 25445549 10.1016/j.tig.2014.09.011

[bib21] Mason A. S. Nelson M. N. Yan G. Cowling W. A., 2011 Production of viable male unreduced gametes in *Brassica* interspecific hybrids is genotype specific and stimulated by cold temperatures. BMC Plant Biol. 11: 103. 21663695 10.1186/1471-2229-11-103 PMC3141635

[bib22] Matsushita S. C. Tyagi A. P. Thornton G. M. Pires J. C. Madlung A., 2012 Allopolyploidization lays the foundation for evolution of distinct populations: evidence from analysis of synthetic *Arabidopsis* allohexaploids. Genetics 191: 535–547. 22426881 10.1534/genetics.112.139295 PMC3374316

[bib23] Pérez-Vich B. Akhtouch B. Munoz-Ruz J. Fernandez-Martinez J. M. Jan C. C., 2002 Inheritance of resistance to a highly virulent race F of *Orobanche cumana* Wallr. in a sunflower line derived from interspecific amphiploids. Helia 25: 137–143.

[bib24] Ramanna M. S. Jacobsen E., 2003 Relevance of sexual polyploidization for crop improvement-a review. Euphytica 133: 3–18.

[bib25] Ramsey J. Schemske D. W., 1998 Pathways, mechanisms, and rates of polyploidy formation in flowering plants. Annu. Rev. Ecol. Syst. 29: 467–501.

[bib26] Ravi M. Marimuthu M. P. Siddiqi I., 2008 Gamete formation without meiosis in *Arabidopsis*. Nature 451: 1121–1124. 18272967 10.1038/nature06557

[bib27] Seiler G. J. Jan C. C., 2014 Wild sunflower species as a genetic resource for resistance to sunflower broomrape (*Orobanche cumana* Wallr.,). Helia 37: 129–139.

[bib28] Singh M. Goel S. Meeley R. B. Dantec C. Parrinello H., 2011 Production of viable gametes without meiosis in maize deficient for an ARGONAUTE protein. Plant Cell 23: 443–458. 21325139 10.1105/tpc.110.079020 PMC3077773

[bib29] Storme N. D. Geelen D., 2013 Sexual polyploidization in plants – cytological mechanisms and molecular regulation. New Phytol. 198: 670–684. 23421646 10.1111/nph.12184 PMC3744767

[bib30] Storme N. D. Mason A., 2014 Plant speciation through chromosome instability and ploidy change: cellular mechanisms, molecular factors and evolutionary relevance. Curr. Plant Biol. 1: 10–33.

[bib31] Tayalé A. Parisod C., 2013 Natural pathways to polyploidy in plants and consequences for genome reorganization. Cytogenet. Genome Res. 140: 79–96. 23751271 10.1159/000351318

