# Supplementary figures and images for: Triploid Production from Interspecific Crosses of Two Diploid Perennial Helianthus with Diploid Cultivated Sunflower (Helianthus annuus L.)
Source: G3 (Bethesda). 2017 Feb 7;7(4):1097–108. doi: 10.1534/g3.116.036327 (PMC5386858; doi:10.1534/g3.116.036327)

## Slide 1
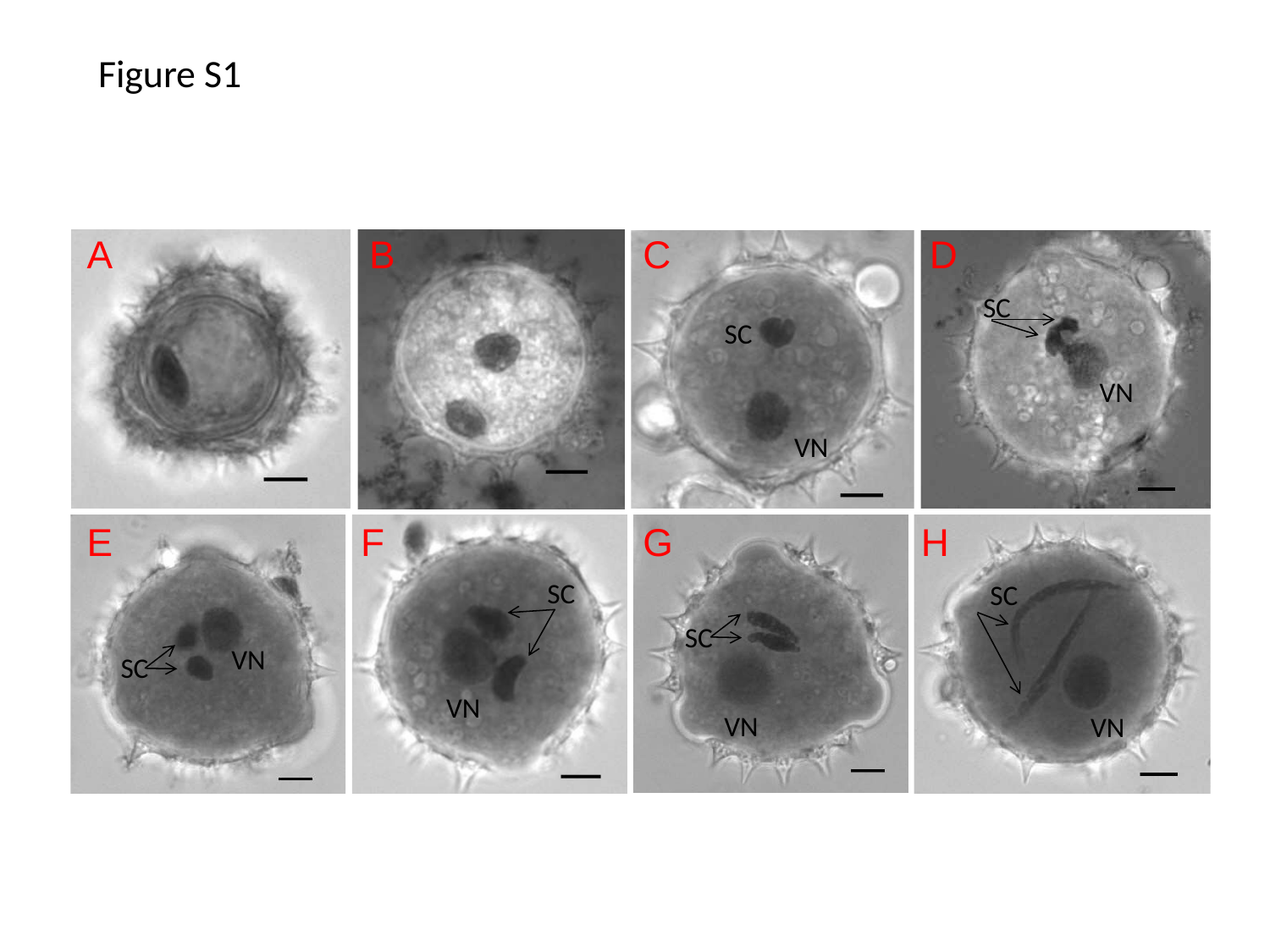

Figure S1
A B C D
SC
SC
VN
VN
E F G H
SC
SC
SC
VN
SC
VN
VN
VN

Supplement: Supplementary file 1 [file 1097FigureS1.pptx]

## Slide 1
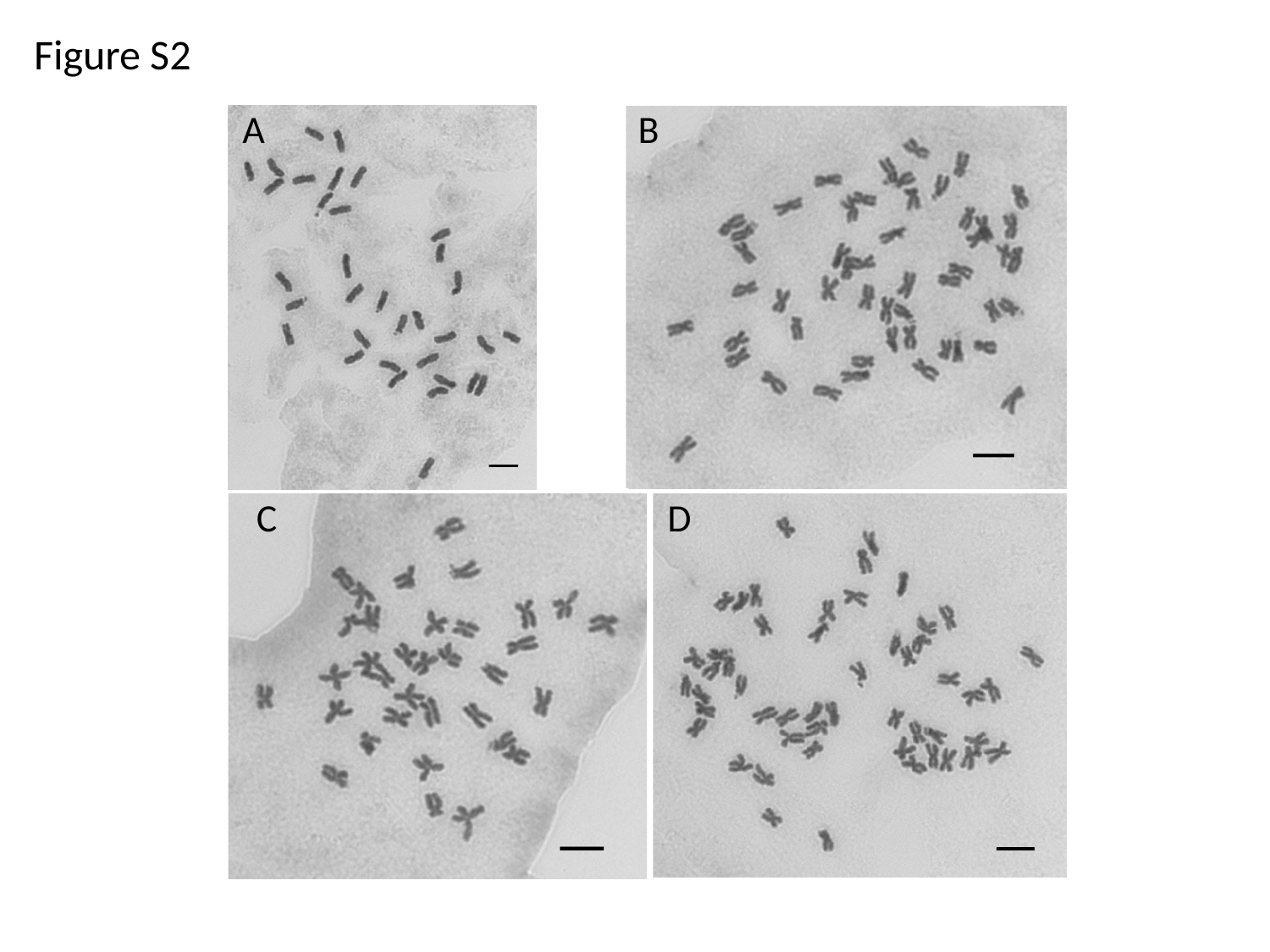

Figure S2
A B
 C D

## Slide 2
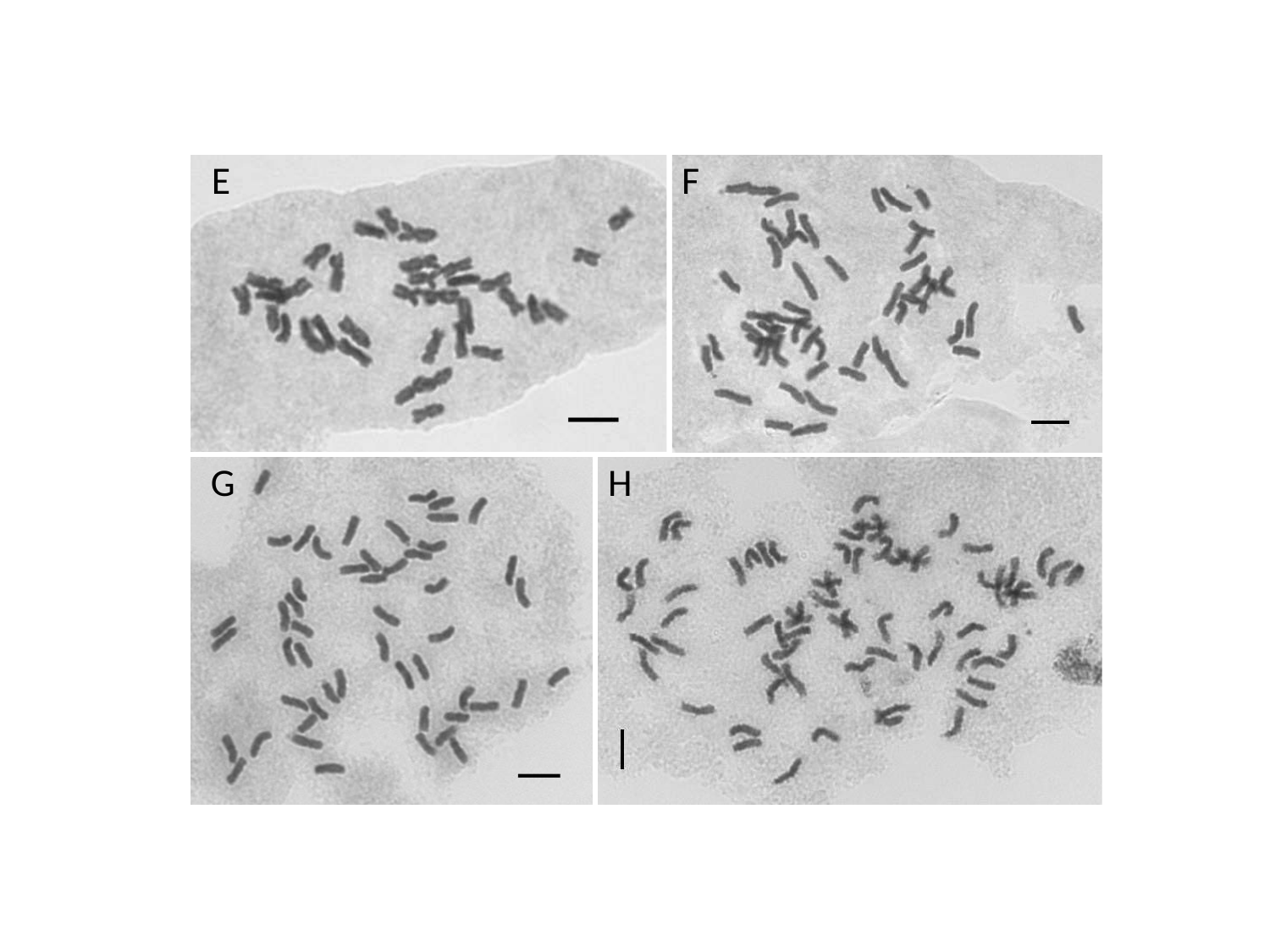

E F
G H

## Slide 3
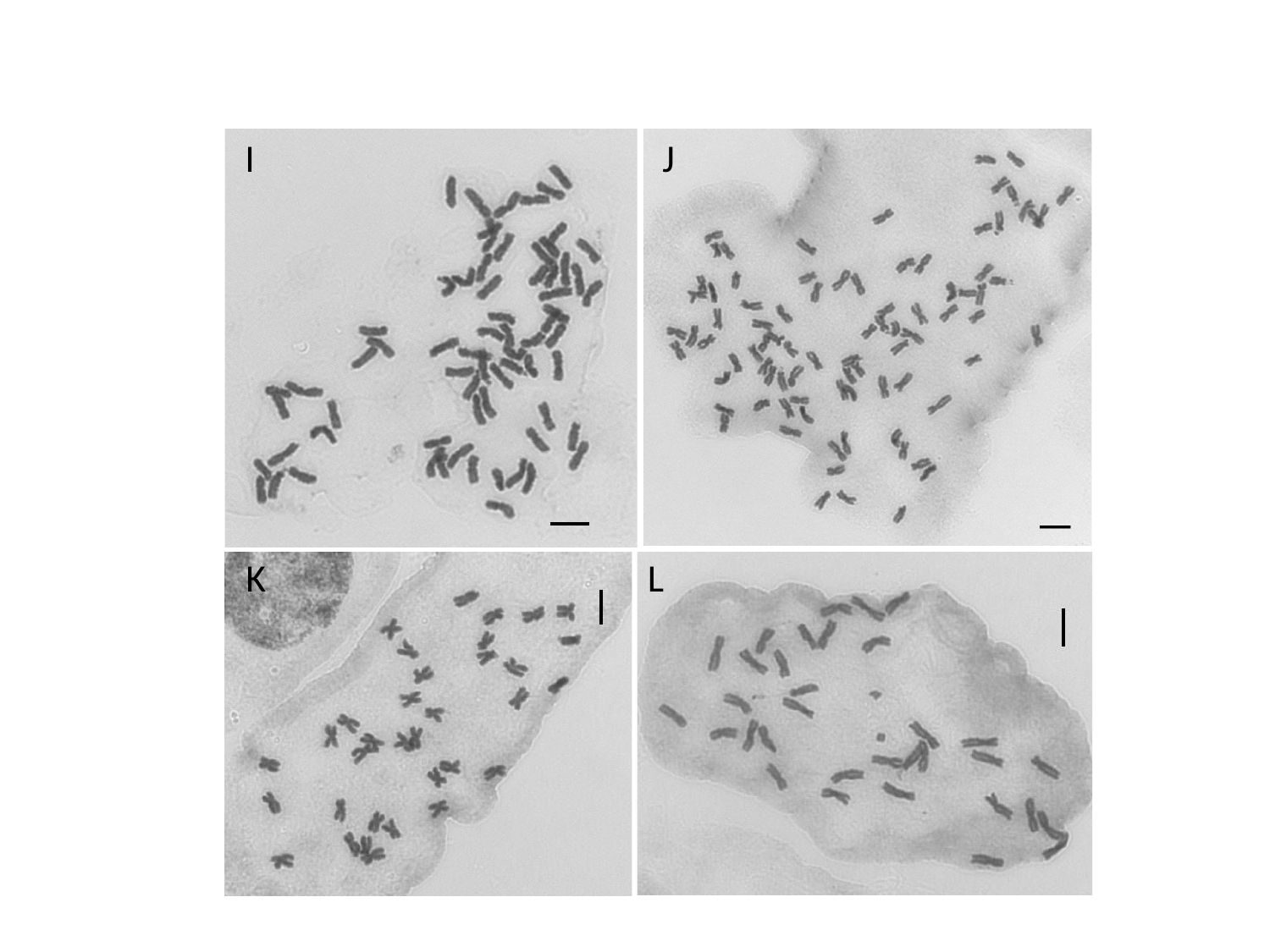

I J
K L

## Slide 4
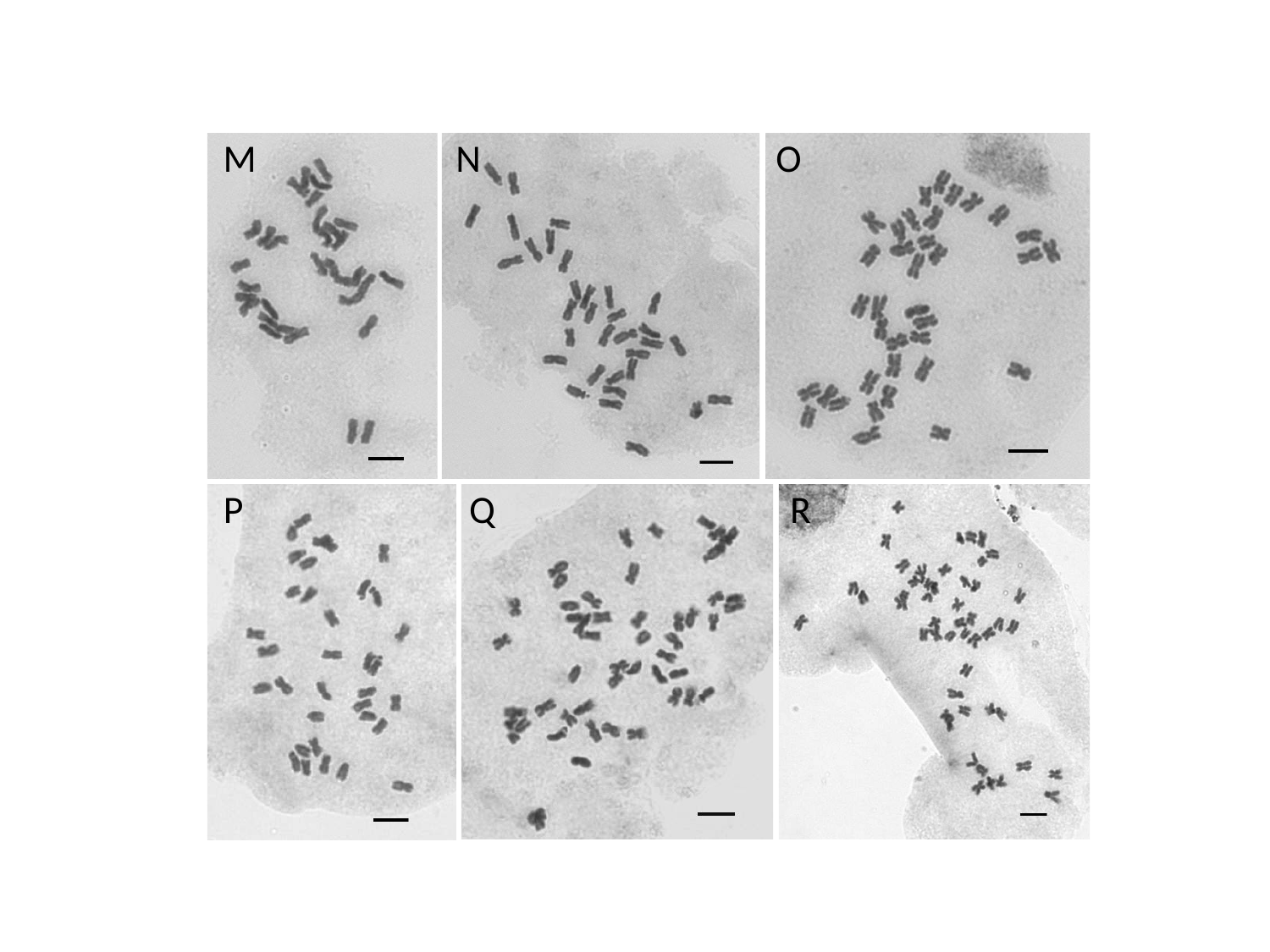

M N O
P Q R

Supplement: Supplementary file 2 [file 1097FigureS2.pptx]
