# Supplementary material for: Triploid Production from Interspecific Crosses of Two Diploid Perennial Helianthus with Diploid Cultivated Sunflower (Helianthus annuus L.)
Source: G3 (Bethesda). 2017 Feb 7;7(4):1097–108. doi: 10.1534/g3.116.036327 (PMC5386858; doi:10.1534/g3.116.036327)
Supplement: Supplementary file 3 [file 1097FigureS3.pptx]

## Slide 1
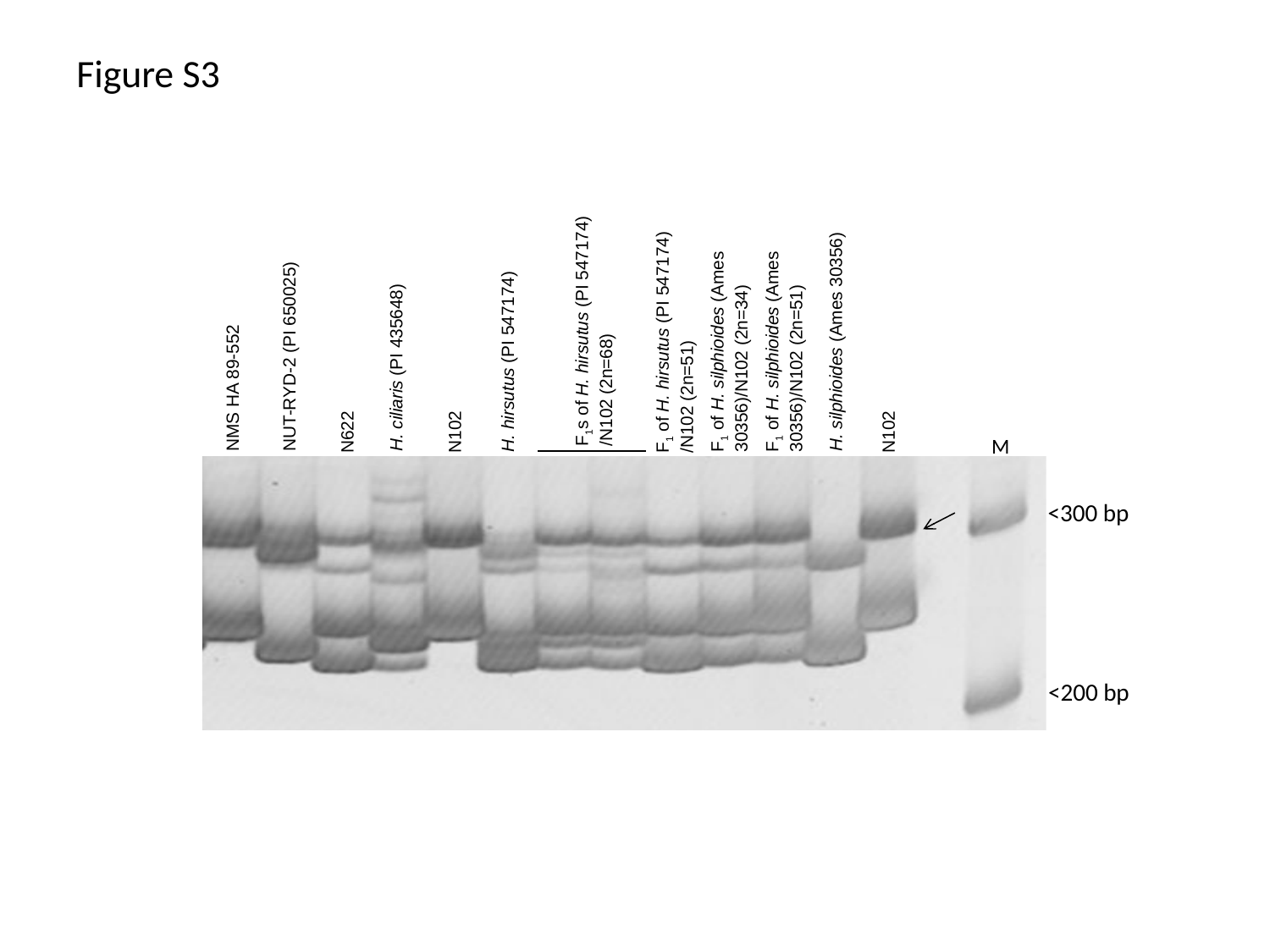

Figure S3
F1s of H. hirsutus (PI 547174)
/N102 (2n=68)
F1 of H. hirsutus (PI 547174)
/N102 (2n=51)
F1 of H. silphioides (Ames 30356)/N102 (2n=51)
F1 of H. silphioides (Ames 30356)/N102 (2n=34)
H. silphioides (Ames 30356)
NUT-RYD-2 (PI 650025)
H. hirsutus (PI 547174)
H. ciliaris (PI 435648)
NMS HA 89-552
N622
N102
N102
M
<300 bp
<200 bp
