# Supplementary material for: Triploid Production from Interspecific Crosses of Two Diploid Perennial Helianthus with Diploid Cultivated Sunflower (Helianthus annuus L.)
Source: G3 (Bethesda). 2017 Feb 7;7(4):1097–108. doi: 10.1534/g3.116.036327 (PMC5386858; doi:10.1534/g3.116.036327)
Supplement: Supplementary file 4 [file 1097FileS1.docx]

**Figure S1.** Pollen mitotic analysis for *H. nuttallii* N102. (A) Polarized microspore. (B and C) Bicellular pollen. (D-H) Different stages of tricellular pollen. VN=vegetative nucleus; SC=sperm cell. Note that the two elongated sperm cells and one vegetative nucleus in Figure H. Bars=5 μm.

**Figure S2.** Chromosome squashes of F_1_ progenies with normal and abnormal chromosome numbers derived from different crosses. (A) Diploid F_1_ of N622/N102 and (B) Triploid N622/N102. (C and D) Diploid and Triploid F_1_ of NUT-RYD-2/N102, respectively. (E and F) Diploid and Triploid F_1_ of *H. silphioides* (Ames 30356)/N102, respectively. (G and H) Triploid and Tetraploid F_1_ of *H. hirsutus* (PI 547174)/N102, respectively. (I) Tetraploid F_1_ of N102/*H. ciliaris* (PI 435648). (J) F_1_ of *H. ciliaris* (PI 435648)/N102 (2n=5x=85). (K) Diploid F_1_ of M1113/N102. (L) An F_1_ of M1113/N102 (2n=2x-1=33). (M-O) Three F_1_s of N102/N314, with 2n=32, 33, and 36, respectively. (P-R) Three F_1_s of NMS HA 89-552/*H. divaricatus* (PI 503216), with 2n=34, 51, and 49, respectively. Bars=5 μm.

**Figure S3.** Polymorphism screening of several *Helianthus* accessions including *H. nuttallii* N102, and confirmation of the F_1_ hybrids using SSR marker ORS505. The arrow indicates the dominant marker in the F_1_ progeny from N102. M: 100-bp plus ladder Gelpilot (Qiagen).
