# Supplementary material for: Triploid Production from Interspecific Crosses of Two Diploid Perennial Helianthus with Diploid Cultivated Sunflower (Helianthus annuus L.)
Source: G3 (Bethesda). 2017 Feb 7;7(4):1097–108. doi: 10.1534/g3.116.036327 (PMC5386858; doi:10.1534/g3.116.036327)
Supplement: Supplementary file 5 [file 1097TableS1.docx]

**Table S1.** The pollen fertility analysis of 22 wild *Helianthus* accessions and HA 89

| **Plant code** | **Species^a^** | **LF %^b^** | **LS %** | **SF %** | **SS %** | **Total Pollen** | **LF+LS %** |
| --- | --- | --- | --- | --- | --- | --- | --- |
| HA 89 | ANN | 0.00 | 0.00 | 95.98 | 4.02 | 1715 | 0.00 |
| G11/1305 | DIV | 0.05 | 0.09 | 98.95 | 0.91 | 2194 | 0.14 |
| LAC28-2 | LAC | 0.16 | 0.16 | 78.49 | 21.19 | 2548 | 0.31 |
| M214 | MAX | 0.00 | 0.00 | 99.12 | 0.88 | 2378 | 0.00 |
| M609 | MAX | 0.00 | 0.13 | 99.21 | 0.66 | 2282 | 0.13 |
| M1113 | MAX | 0.52 | 0.33 | 90.89 | 8.26 | 1537 | 0.85 |
| 30MOL-2 | MOL | 0.00 | 0.00 | 97.31 | 2.69 | 2194 | 0.00 |
| MOL A 3201 | MOL | 0.32 | 0.37 | 72.88 | 26.43 | 1899 | 0.68 |
| N102 | NUT | 1.22 | 0.79 | 95.18 | 2.80 | 1640 | 2.01 |
| N622 | NUT | 0.00 | 0.00 | 93.42 | 6.58 | 1626 | 0.00 |
| N902 | NUT | 0.00 | 0.00 | 98.01 | 1.99 | 1860 | 0.00 |
| N903 | NUT | 0.00 | 0.00 | 97.89 | 2.11 | 1563 | 0.00 |
| NUT-RYD-2 | NUT | 0.00 | 0.00 | 99.25 | 0.75 | 1867 | 0.00 |
| N314 | NUT | 0.17 | 0.09 | 97.61 | 2.13 | 2346 | 0.26 |
| N412 | NUT | 0.00 | 0.00 | 99.52 | 0.48 | 2281 | 0.00 |
| N405 | NUT | 0.23 | 0.00 | 98.37 | 1.40 | 2646 | 0.23 |
| N424 | NUT | 0.00 | 0.04 | 89.54 | 10.41 | 2314 | 0.04 |
| N817 | NUT | 0.05 | 0.00 | 55.17 | 44.78 | 1934 | 0.05 |
| PUM24-1 | PUM | 0.00 | 0.05 | 38.92 | 61.03 | 2148 | 0.05 |
| PUM24B | PUM | 0.09 | 0.00 | 45.12 | 54.78 | 2143 | 0.09 |
| RES28382 | RES | 0.05 | 0.00 | 99.53 | 0.43 | 2116 | 0.05 |
| RES28386 | RES | 0.00 | 0.05 | 99.32 | 0.62 | 1922 | 0.05 |
| CIL29-3 | CIL | 0.46 | 0.07 | 98.48 | 0.99 | 1516 | 0.53 |

a: The first three letters of the *Helianthus* species are used to identify the species.

b: LF=large fertile, LS=large sterile, SF=small fertile, and SS=small sterile.
